# Improvements of acute flaccid paralysis and measles surveillance performances in response to outbreak of circulating vaccine-derived poliovirus (2021-2022): the case of Southwest Ethiopia Region, Ethiopia

**DOI:** 10.11604/pamj.2024.49.23.37746

**Published:** 2024-09-27

**Authors:** Amenu Wesen Denegetu, Tadesse Gossaye Birru, Eshetu Wassie Asemahaegn

**Affiliations:** 1 World Health Organization, Expanded Programme on Immunization, Maternal Child Health and Nutrition, Addis Ababa, Ethiopia,; 2 World Health Organization, Expanded Programme on Immunization, Maternal Child Health and Nutrition, Islamabad, Pakistan

**Keywords:** Polio eradication, non-polio AFP rate, non-measles febrile rash, acute flaccid paralysis, measles

## Abstract

**Introduction:**

following the detection of vaccine-derived poliovirus in 2019 in Ethiopia, response activities have been conducted including strengthening disease surveillance activities.

**Methods:**

trend analysis study design of acute flaccid paralysis and measles surveillance data for the years 2021 and 2022 for Southwest Ethiopia Region was used. The non-polio acute flaccid paralysis (AFP) rate and stool adequacy rates were used to assess the AFP surveillance. Whereas the non-measles febrile rash rate was used to assess the measles surveillance.

**Results:**

a total of 68 AFP cases in 2022 and 49 in 2021 have been reported as of week 41 and investigated for polio analysis. All cases were discarded in 2022 and 1 cVDPV was detected in 2021. The stool adequacy rate for 2022 was 96%; whereas, 94% in 2021. The annualized non-polio AFP rate was 4.8/100,000 for 2022 and 3.8/100,000 for 2021, which the former is much higher though both met the minimum expected rate in outbreak areas of 3/100,000. A total of 155 suspected measles cases in 2022 and 38 in 2021 have been investigated for IGM analysis. In 2022, 9 and 1 in 2021 Igm positive for measles were identified. The non-measles febrile rash rate for 2022 was 4.6/100,000; whereas, 1.2/100,000 for 2021.

**Conclusion:**

there is an improvement in the sensitivity of AFP and measles surveillance for Southwest Ethiopia Region in 2022. Sustaining high-quality measles and AFP surveillance is suggested to maintain measles and polio-free statuses.

## Introduction

Southwest Ethiopia is one of the 13 provinces/regions in Ethiopia. The region has an estimated total population of 3.3 million (2022), i.e.; 3% of the national population. It is divided into 6 zones and 53 districts. Bonga town is the seat of regional government and the largest city in the region. There is one teaching hospital, four general hospitals, and 123 health centers. The backbones of vaccine-preventable diseases (VPD), including AFP and measles, surveillance in the region are district health offices; which are dependent on their respective health facilities; i.e. health centers (HCs) and hospitals (Hosps). Each HCs or Hosp has designated surveillance focal persons who should have basic training on disease surveillance, sometimes called integrated diseases surveillance and response focal persons (IDSR FP). Each district/*woreda* has a public health emergency management focal person (PHEM FP), who coordinates all surveillance sites in the respective district. *Woreda* PHEM FPs have a surveillance structure, which lists all health facilities (both for-profit and nonprofit) and community informant sites. Hence, all suspected diseases of surveillance interest that occur at any of the regular health care systems or in one of the community sites, including rumors, will be reported to *woreda* PHEM FP.

Then the FP will verify if it satisfies the case definitions of interest, and case investigation and specimen collection will be done while being reported to the provincial level for line-listing for further analysis, interpretation, and communication for appropriate and timely response interventions. Following a circulating vaccine-derived poliovirus type 2 (cVDPV2) outbreak in 2019 in Somali Region of Ethiopia [1], nationwide emergency response activities have been in place including strengthening routine immunization activities, supplementary immunization activities, capacity building of surveillance officers, sensitization of the general health staff and strengthening disease surveillance activities. Hence, well-documented improvements were achieved in Southwest Ethiopia Region following integrated efforts by polio eradication partners. This improvement should be endorsed to sustain the performances that accelerate the road to global polio eradication [2]. The key objective of measles surveillance is to identify areas of measles virus transmission and immunity gaps. This will guide effective public health responses to achieve the elimination of endemic measles and sustain elimination in post-elimination settings [3]. This study aims to assess improvements in AFP and measles surveillance indicators of Southwest Ethiopia Region in response to the polio outbreak at week 41 of 2022 compared with the same time period of 2021.

## Methods

**Study design and setting:** a trend analysis study design of acute flaccid paralysis (AFP) and measles surveillance data received from all *woredas*/districts of Southwest Ethiopia Region was used to assess the improvements in the AFP/measles surveillance performance after the polio outbreak for the same time period of week 41 of 2022 and 2021. The review of weekly AFP and Measles surveillance performance key indicators was used from week 1 to week 41 of 2022 and 2021. Key AFP and measles surveillance indicators were used for all cases which had been investigated and specimens collected for testing in the measles or polio laboratories. This was to compare the trend of sensitivity of the surveillance systems over time in the two years. Confirmed weekly update information from the national Polio and measles control data center was processed for the same period to understand the performances of key indicators. The weekly reported AFP and measles surveillance performances were compared for the two years. The differences were subjected to statistical tests for ascertaining levels of significance.

**Study population:** all reported AFP and suspected measles cases from Southwest Ethiopia Region at week 41 of 2022 and 2021 are the study population. Hence, the cumulative AFP and suspected measles cases reported at week 41 of 2021 were compared with the cumulative caseloads of suspected measles and AFP cases reported at the same time period of 2022 from all districts of Southwest Ethiopia Region, Ethiopia. The World Health Organization (WHO) standard case definitions for both AFP and suspected or confirmed measles case definitions were used for this study. An AFP case is defined as a child less than 15 years of age with sudden onset of floppy paralysis or weakness of any of the limbs, irrespective of the causes. Whereas, a suspected measles case is defined as any person of any age with fever, maculopapular rash and cough or conjunctivitis or coryza (the 3C); whereas a confirmed measles case is laboratory confirmed case, i.e., a suspected measles case with measles IgM positive. All AFP cases with, if any, circulating vaccines derived polioviruses and those reported as compatible with polio were all included in the study. Those AFP cases classified as “not AFP” were excluded from this study. Besides, those suspected measles cases who were investigated and blood specimens collected for laboratory analysis were only included in this study.

**Data collection and analysis:** we retrieved the weekly AFP Epidemiological update of week 41 of both 2021 and 2021, which is compiled by National Polio Eradication Initiative (nPEI) data control room of WHO Ethiopia, to get the line-listing of all AFP cases reported from Southwest Ethiopia Region [4,5]. Similarly, data for measles surveillance trend was retrieved from Measles and Neonatal Tetanus weekly update of week 41 2022 and 2021, which is compiled by national measles elimination control room, WHO Ethiopia [6,7]. The data obtained from the nPEI (ETHIOPIA) control room for AFP and measles surveillance was directly used for this analysis. In addition, semi analyzed data for key indicators for both AFP and Measles were directly utilized for this study. Both AFP and measles surveillance data for Southwest Ethiopia Region were filtered for the purpose of this study. Data were analyzed manually and presented in tables accordingly on the selected thematic areas.

**Definitions and key parameters:** the WHO main surveillance indicators were used as parameters to assess the changes over time on AFP and measles surveillance systems. The scope of parameters covered from investigation of the reported AFP and suspected measles cases to the release of final laboratory results by the national polio lab and/or provincial measles laboratories. The non-polio AFP rate (NP-AFP) and stool adequacy rate were mainly used to assess the sensitivity of the AFP surveillance systems. Whereas the non-measles febrile rash rate (NMFR) was mainly used to assess the sensitivity of measles surveillance system. The stool adequacy of reported AFP cases was measured by the percentage of AFP cases investigated with two stool specimens collected within two weeks of onset of weakness or paralysis. On the other hand, the NP-AFP rate was measured by the number of non-polio AFP cases detected per 100,000 under 15 population in a certain district or region. In addition, the completeness of 60-day follow-up examination was also used to assess the percentage of follow-up examinations conducted for all inadequate AFP cases within two months of onset of paralysis.

**Ethical considerations:** the study proposal has been approved by the regional ethics and research review committee. Besides, the results do not mention any individual personal identifications and would not harm any study participant except that the results will help to improve disease surveillance activities in the regional state and in the country as well.

## Results

**General characteristics of study participants:** in 2022, a total of 68 AFP cases and 155 suspected measles cases were reported from the region as of week 41 of 2022. Of the AFP cases, 39 cases were below 5 years, and 29 cases were 5-15 years. In 2021, a total of 49 AFP cases and 39 suspected measles cases were reported from the region as of week 41 of 2021. Of the AFP cases, 31 cases were <5 years and 18 cases were 5-15 years (Table 1, Table 2).

**Table 1:** acute flaccid paralysis surveillance performance indicators as of week 41 for 2021 and 2022, Southwest Ethiopia Region, Ethiopia

Year	Total cases	Adequate cases	Stool adequacy rate (%)	Annualized NPAFP rate per 100,000	Wild/CVDP cases	Overall performance status
2021	49	46	94	3.8	1	High NPAFP and High Stool adequacy rate
2022	68	65	96	4.8	0	High NPAFP and High Stool adequacy rate

NPAFP: non-polio acute flaccid paralysis; CVDP: Circulating Vaccine-Derived Polio

**Table 2:** measles surveillance performance indicators as of week 41 for 2021 and 2022, Southwest Ethiopia Region, Ethiopia

Year	Total cases with blood specimen	Adequate cases	Adequacy rate (%)	Annualized NMFR rate per 100,000	Measles IGM +	Measles detection rate	Incidence rate per 100,000
2022	155	108	100	4.6	9	4.9	30
2021	38	148	100	1.2	1	1.4	1

NMFR: non-measles febrile rash; IGM: Immunoglobulin M

**Key performance indicators for AFP surveillance, comparing 2022 with 2021:** in 2022 as of week 41, a total of 68 AFP cases have been detected. All cases were discarded and no wild or vaccine-derived polioviruses were identified. The stool adequacy rate was 95.6%. The NP-AFP rate for the period was 4.8/100,000, which is more than the minimum rate expected in outbreak areas, 3/100,000. Hence, the regions´ overall performance is at high NP-AFP rate and high stool adequacy. In 2021 as of week 41, a total of 49 AFP cases have been detected. All cases were discarded, and one vaccine-derived poliovirus was identified. The stool adequacy rate was 93.9%. The NP-AFP rate for the period was 3.8/100,000. Hence, the regions´ overall performance is at high NP-AFP rate and high stool adequacy (Table 1).

**Key performance indicators for measles surveillance, comparing 2022 with 2021:** in 2022 as of week 41, a total of 155 suspected measles cases with blood specimens have been reported. A total of 9 confirmed IGM positive for a total of 38 suspected measles cases with blood specimens cases identified. One hundred and sixteen measles cases were epidemiologically linked to previous outbreaks in the region and no measles compatible cases identified. Specimen adequacy rate was 100% and overall measles detection rate for the region was 4.9%. The NMFR rate for the study period was 4.6/100,000. In 2021 as of week 41, a total of 38 suspected measles cases with blood specimens have been reported. One confirmed IGM positive for measles case was detected and no rubella IGM positive case identified. Specimen adequacy rate was 100% and overall measles detection rate for the region was 1.4. The NMFR rate for the period was 1.2/100,000 (Table 2). The non-polio enterovirus isolation rate for both 2022 and 2021 was below 10%; i.e., 4.4% for 2022 and 6.1% for 2021.

## Discussion

The study assessed improvements of AFP and measles surveillance indicators of Southwest Ethiopia Region in response to the 2019 national polio outbreak as of week 41 of 2022 compared with the same time period of 2021. The finding revealed that, both AFP surveillance and measles surveillance indicators had been improved drastically as indicated in the result section of the study. An increased in the number of reported AFP cases in 2022 was clearly indicated and attributed to enhanced surveillance activities conducted following the CVDP outbreak in the country including the detection of a case in the region of study. Following the outbreak, response activities have been conducted; including national nOPV2 immunization campaign, at which time, strengthening of active case search, surveillance training, capacity building of health staffs with provision of specimen collection and investigation materials, facilitation of specimen collection, packaging and transportation to national laboratory and sessions of clinician sensitization have been conducted. Besides, with better response lessons, directions and recommendations from the global health regulators [7] integrated COVID-19 and routine surveillance activities has resulted in marked improvements in all performance indicators of both AFP and measles surveillance in 2022.

In addition, the WHO interim guidelines of continuation of the immunization services and diseases surveillance were implemented by countries [8,9]. On the other hand, the decrease in the number of AFP cases in 2021 was mainly attributed to COVID-19 epidemiology and associated restrictions on movement of polio staff members and diversion of some resources from polio to the COVID-19 response [10]. The NP-AFP rate for 2022 has improved significantly to 4.8/100,000 compared with 3.8/100,000 in 2021, an improvement of 26.3%, which shows the surveillance became more sensitive and would not miss any polio outbreaks in the region. The findings of NP-AFP rate in both 2022 (4.8) and 2021 (3.8) meet the surveillance indicator standard in outbreak setting, i.e. >3 NP-AFP rate. According to the Polio weekly update, Ethiopia, Week 51, 2021 update, there were 6 VDPV2, 12 cases of c-VDPV2 and 1 environmental cVDPv2 in Ethiopia including in one bordering Region (Oromia) to Southwest Ethiopia [11]. A report by MMWR, during January 2020 - June 2021, there were 38 cVDPV2 emergences in active transmission in 34 countries; 28 (82%) of these countries are in Africa including Ethiopia [12].

The progress towards the global polio eradication has been bumped after a recent polio outbreak in Africa whereby the health authorities of Malawi have declared an outbreak of wild poliovirus type 1 after a case was detected in a young child in the capital Lilongwe. This is the first case of wild poliovirus in Africa in more than five years after Africa was declared free of indigenous wild polio in August 2020 [13]. Studies show that interruptions to poliovirus surveillance might have negative consequences on detection of poliovirus circulation and hence continuous analysis of AFP reporting trends is necessary to better understand the long-term impact to the eradication initiative [14]. The improvement in case detection in 2022 can also be attributed to the deployment of polio consultant for the region by global polio eradication initiative partners, strengthening capacity of local health staff through training and supportive supervision. Besides, the stool adequacy rate has improved slightly from 94% to 96%, showing a 2.1% improvement. This progress is also attributed similarly to the NP-AFP rate improvement explained above. In both 2022 and 2021, males were more likely affected by AFP than females, with significant proportion of around 60.3% in 2022 and 55.1% in 2021. This finding is similar to results in Nigeria, Ghana, Iran, Italy and India in which higher frequency of AFP was observed among boys than girls [15-17].

Male majority in the incidence of symptomatic infectious diseases in children may be due to reduced immunity, effect of sex hormones, genetic influence or exposure-connected reasons, which clearly need further study. Quite majority (nearly 90%) of the AFP cases developed lower limb (s) paralysis in both 2022 and 2021. This finding is similar to the study finding in East and Southern African countries from 2012-2019 [18]. The non-polio enterovirus isolation rate for both 2022 and 2021 was below 10%, indicating the quality of specimen collection, packaging and transportation seem unsatisfactory. This finding is similar with a study in Palestine where it revealed a 4% NPEV isolation rate [19]. The NMFR rate indicates the sensitivity of the surveillance in detecting suspected measles cases in an area, and the minimum surveillance target is 2/100,000 population [20]. In this study, a total of 155 suspected measles cases with blood specimens were reported as of week 41 of 2022; whereas, in 2021 same period, 38 cases with specimens had been reported; showing an increased in case load of four-fold. The NMFR rate, which is the prime performance indicator for measles surveillance has been significantly improved to make the sensitivity of the surveillance more indicative of early outbreaks in the region. Maintaining high quality measles and AFP surveillance together with high immunization coverage in the region are mandatory strategies to maintain measles and polio free statuses. In addition, sensitive surveillance indicators help to identify quickly importation of polioviruses and detect measles cases rapidly and help for early warning and response interventions accordingly. This study used original data that is officially recognized by national offices, and hence has trustworthy, this can be taken as the strength of the study. Whereas, the findings may be limited to only one region, i.e. nearly 3% of the country´s total population and hence may not be generalized to the whole country.

## Conclusion

There is an improved in the sensitivity of AFP and measles surveillance for Southwest Ethiopia Region in 2022. Both the NP-AFP rate and stool adequacy, the two most important surveillance indicators for AFP surveillance, are achieved above the global minimum expected figure; 4.8/100,000 and 96%, respectively. The global measles elimination target is to achieve 2/100,000, for which the finding in this study for Southwest Ethiopia Region is 4.6/100,000. This indicates the existence of sensitive marker to detect any outbreaks in the region, which again needs to be maintained.

### What is known about this topic

- Achieving high AFP surveillance standards (>2.0 NPAFP rate and > 80% of stool adequacy rate) are key indicators for polio eradication; this study revealed a 4.8 NPAFP rate and 96% stood adequacy rate;
- Achieving high measles surveillance standards (>2.0 NMFR rate) is one of the key indicators for global measles elimination; in this study a 4.6/100,000 NMFR rate was found, which shows the surveillance is very sensitive.

### What this study adds

- As a new region in the country, the findings may be used as a baseline data to be used as a baseline or reference for future performance monitoring;
- The improvement in both AFP and measles surveillance can be taken as a good lesson for other Regions of the country;
- The study may initiate more detailed follow-up study in the new Region.
